# Effects of Fuzheng Huayu recipe on entecavir pharmacokinetics in normal and dimethylnitrosamine-induced hepatic fibrosis rats

**DOI:** 10.1080/13880209.2019.1687527

**Published:** 2019-12-18

**Authors:** Tao Yang, Tian-Hui Zheng, Qiang Zhao, Wei Liu, Shu-Ping Li, Yan-Yan Tao, Chang-Hong Wang, Cheng-Hai Liu

**Affiliations:** a Institute of Liver Diseases, Shuguang Hospital Affiliated to Shanghai University of Traditional Chinese Medicine, Shanghai, China; b Department of Cardiology, Institute of Cardiovascular Disease of Integrated Traditional Chinese Medicine and Western Medicine, Shuguang Hospital affiliated to Shanghai University of Traditional Chinese Medicine, Shanghai, China; c Shanghai Key Laboratory of Traditional Chinese Clinical Medicine, Shanghai, China; d Institute of Chinese Materia Medica, Shanghai University of Traditional Chinese Medicine, Shanghai, China; e The MOE Key Laboratory for Standardization of Chinese Medicines and The SATCM Key Laboratory for New Resources and Quality Evaluation of Chinese Medicines, Shanghai, China

**Keywords:** Anti-hepatitis B virus, Chinese herbal compound, anti-hepatic fibrosis

## Abstract

**Context:**

Fuzheng Huayu recipe (FZHY) combined with entecavir (ETV) is used to treat the cirrhosis caused by chronic hepatitis B (CHB) infection.

**Objective:**

To investigate the effect of FZHY on ETV pharmacokinetics under different conditions.

**Materials and methods:**

A model of liver fibrosis was created by intraperitoneal injection of dimethylnitrosamine (DMN; 10 μg/kg) for 4 weeks in Wistar rats. Ultra-high-performance liquid chromatography–tandem mass spectrometry was used to determine the blood concentration of ETV. Pharmacokinetic characteristics of ETV (0.9 mg/kg) were investigated after co-administration with FZHY (0.55 g/kg) at certain time intervals in normal and model rats.

**Results:**

The analytical method for ETV was validated at 0.5–50 μg/L with a correlation coefficient = 0.9996, lower limit of quantitation of 0.5 μg/L and mean accuracy of 104.18 ± 9.46%. Compared with the ETV-N group, the pharmacokinetic parameters of the EF-2 group did not change significantly, but that of the EF-0 group decreased in *C*_max_ to 27.38 μg/L, in AUC_0–_*_t_* from 323.84 to 236.67 μg/h/L, and a delay in *T*_max_ from 0.75 to 6.00 h; that of the EF-0 group presented a decrease in *C*_max_ of 61.92%, delay in *t*_1/2_ of 2.45 h and delay in *T*_max_ of 2.92 h. The *t*_1/2e_ and *V*_d_/*F* of ETV were increased significantly to 8.01 h and 24.38 L/kg in the ETV-M group.

**Conclusions:**

The effects of FZHY on ETV pharmacokinetics were diminished with an increase of interval time. The best time to administer both drugs is >2 h apart.

## Introduction

**Figure 1.**
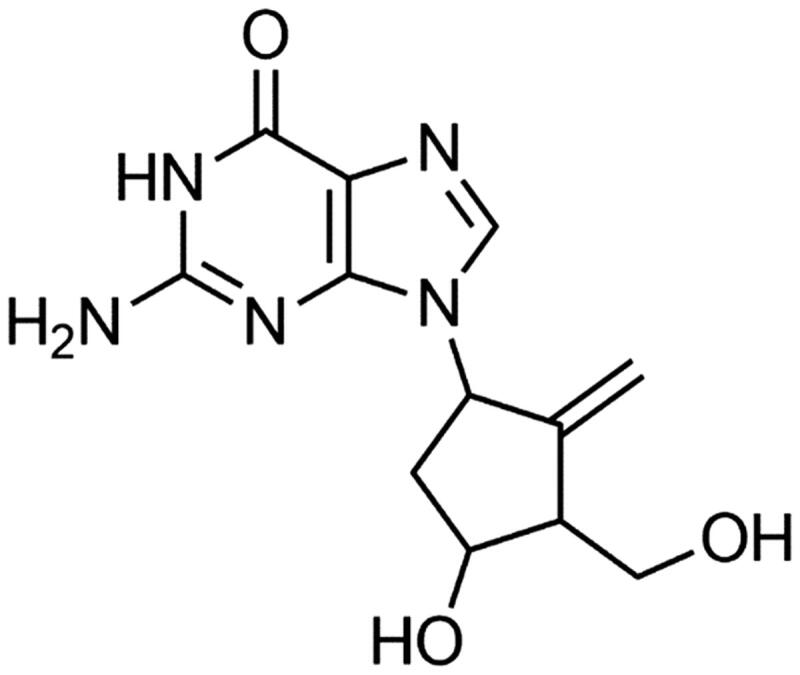
Chemical structure of entecavir (ETV).

Entecavir (ETV), which is a cyclopentanone acid guanosine analogue (Figure 1), efficiently inhibits HBV replication through the introduction of frequent viral polymerase mutations, and can significantly improve liver function in the treatment of hepatitis B virus (HBV) (Lai et al. 2002; Papatheodoridis et al. 2018). Currently, it has become the first-line treatment recommended for adults with chronic hepatitis B (CHB) (Pawlotsky 2009). However, ETV: (i) may have drug resistance and viral rebound after withdrawal; (ii) can inhibit replication of only HBV; has a limited therapeutic effect on liver fibrosis (Ahn et al. 2013). Therefore, finding a therapeutic regimen that can simultaneously control HBV replication and improve hepatic fibrosis is needed.

Fuzheng Huayu recipe (FZHY) is an anti-fibrotic agent approved by the China Food and Drug Administration (CFDA), and has been used widely for treating liver fibrosis caused by CHB infection (Yang, Liu, Wang, et al. 2015; Yang, Liu, Zheng, et al. 2015). Pharmacological studies and clinical trials have demonstrated that FZHY has a significant effect against liver fibrosis because it can inhibit activation of hepatic stellate cells, protect hepatocytes and inhibit hepatic sinusoidal capillarization (Liu et al. 2009). Furthermore, it has been reported that ETV combined with FZHY has a good synergistic effect for the treatment of liver fibrosis due to HBV infection, and can reduce the prevalence of adverse reactions (Suqiu et al. 2013). Studies have shown that ETV is not an inhibitor or inducer of cytochrome P450 in humans (Palumbo 2008), but an inhibitor of some transporters: organic anions (OATs), cations (OCTs) and concentrative nucleoside (CNTs) (Mandíková et al. 2016). Furthermore, *Salvia miltiorrhizae* (Danshen), an important ingredient of FZHY, has been demonstrated to elicit significant competitive inhibition on OAT1- and OAT3-mediated substrate uptake in humans (Wang and Sweet 2013). Conversely, it has been reported that food affects the absorption of ETV, and leads to decreases in maximum concentration (*C*_max_) and area under the concentration–time curve (AUC) (Zhang et al. 2010). Hence, it is recommended that ETV is administered on an empty stomach (≥2 h before or after meals) (Matthews 2006). Based on these studies, it can be inferred that the pharmacokinetics of ETV may be affected by FZHY when taken together.

We developed a rapid and sensitive ultra-high-performance liquid chromatography–tandem mass spectrometry (UHPLC–MS/MS) method to determine the ETV concentration in rat plasma. The method was applied to study the effect of FZHY on ETV pharmacokinetics in normal rats and rats suffering from hepatic fibrosis after oral administration of ETV at different time intervals. We aimed to provide a meaningful basis for developing a clinical dose regimen for the treatment of hepatic fibrosis by combination of ETV with FZHY.

## Materials and methods

### Chemicals and reagents

ETV (purity >98%) was purchased from the Shanghai Shifeng Biological Technology Co., Ltd. (Shanghai, China); ETV tablets were purchased from the local pharmacy as Baraclude (Bristol Myers Squibb, New York, NY, USA); dimethylnitrosamine (DMN) was purchased from Tokyo chemical industry Co., Ltd (Tokyo, Japan); osalmid (purity >98%) was purchased from Sigma-Aldrich Co. (St. Louis, MO); heparin sodium was purchased from Sinopharm Chemical Reagent Co., Ltd. (Shanghai, China); HPLC grade acetonitrile and methanol were purchased from Fisher Scientific (Fisher, Waltham, MA, USA). Formic acid (purity >99%) was purchased from Roe Scientific Inc. (Newark, NJ, USA). All the other reagents were of analytical grade. Deionized water was purified using a Milli-Q system (Millipore, Billerica, MA, USA). Kits used for biochemical analyses were purchased from Nanjing Jancheng Bioengineering Institute (Nanjing, China).

FZHY recipe powder (Fuzheng Huayu capsule contents, lot #140616) was prepared and provided by Shanghai Sundise Medicine Technology Development Co. Ltd. (Shanghai, China) (CFDA approval no.: z20050546). The quantitative analysis of FZHY recipe powder has been published previously (Wang et al. 2010; Yang et al. 2013). According to human dosage in clinical practice and human-rat coefficient of skin surface area, FZHY powder was suspended in distilled water, and administered orally at a dose of 0.55 g/kg (human equivalent dose).

### Animals and model preparation

Forty-eight male Wistar rats (weight: 120–140 g) were purchased from Beijing Vital River Laboratory Animal Technology Co., Ltd. (license number: SCXK (Jing) 2012-0001). Animals were maintained at standard room temperature (22 ± 2 °C) and relative humidity (60 ± 5%) with a 12 h light/dark cycle with food and water available *ad libitum*. All experimental procedures were approved by the Institutional Animal Care and Use Committee of Shanghai University of Traditional Chinese Medicine.

Forty-eight male Wistar rats were divided into two groups: control (*n* = 30) and model (*n* = 18). Rats in the model group were injected with DMN (10 mg/kg body weight, i.p.) every other day for 4 weeks according to the method of Yang et al. (2015). At the end of modelling, six rats for each group were killed and used for biochemical and pathology studies. Blood samples were obtained from the inferior vena cava, and centrifuged at 3000 rpm for 30 min at 4 °C. After 3 h, the serum was maintained at –70 °C for liver function tests. The livers were removed rapidly. Liver tissue was taken from the right lobe, fixed in 10% phosphate-buffered formaldehyde, and processed for embedding in paraffin. The remainder of the rats were employed for the pharmacokinetics research.

### Analyses of serum transaminase activity and histology of liver sections

Serum levels of alanine aminotransferase (ALT) and aspartate transaminase (AST) were determined with standard kits according to the manufacturer instructions. Liver tissues were fixed with 10% formalin, embedded in paraffin and cut into 4 μm sections for staining with haematoxylin and eosin (H&E). For quantitative assessment of fibrosis, sections were stained with Sirius Red for collagen deposition. Fibrosis was staged as: stage 0, no fibrosis; stage 1, expansion of the portal tracts without linkage; stage 2, portal expansion with portal-to-portal linkage; stage 3, extensive portal-to-portal and focal portal-to-central linkage; stage 4, cirrhosis (Scheuer 1991). The area of collagenous fibres was quantified by imaging analysis.

### Apparatus and operating conditions

#### Liquid chromatography

The Agilent 6410 Triple Quad UPLC-MS/MS system (Agilent Technologies Inc., Lexington, MA) equipped with an Agilent 1290 UPLC system (Agilent Inc., Lexington, MA) consisting of a binary pump (G4220A), an autosampler (G4226A), a degasser (G1330B) and an automatic thermostatic column (G1316C) compartment was used for the experiments. All measurements were carried out with the mass spectrometer operated in positive electrospray ionization (ESI) mode. All data were processed using Agilent MassHunter Qualitative Analysis B.04.00 Software (Agilent Technologies Inc., Lexington, MA).

Chromatographic separation was achieved on an ACQUITY UPLC HSS T3 column (2.1 mm × 100 mm, 1.8 μm) maintained at 40 °C with a flow rate set at 0.3 mL/min. A gradient elution programmer was used for chromatographic separation with mobile phase A (0.1% formic acid in water) and mobile phase B (acetonitrile) mixed as follows: 0–2.0 min (5–15% B); 2.0–2.5 min (15–30% B); 2.5–3.2 min (30–90% B); 2–4.2 min (90% B); 4.2–4.5 min (90–98% B); 4.5–4.6 min (98–5% B); 4.6–6 min (5% B). The flow rate of the mobile phase was set at 0.3 mL/min and the injection volume was 5 μL.

#### Mass spectrometric conditions

MS/MS was operated in positive mode using the following operating parameters: gas temperature, 325 °C; gas flow, 10 L/min; nebulizer, 35 psi; capillary voltage, 4.0 kV; fragmentor voltage, 130 V; cell accelerator, 3 V; and dwell, 200 ms. Quantification transitions were set at *m/z* 278.2 → *m/z* 152.1 with fragmentation energy (FE) of 120 V and collision energy (CE) of 15 eV for ETV and *m/z* 230.0 → *m/z* 121.2 with FE of 120 V and CE of 15 eV for Osalmid as the internal standard (IS).

#### Preparation of stock solution, calibration and quality control samples

The stock solution of ETV (0.2 μg/mL) was prepared by dissolving the required amount of the reference standard in methanol and then diluted using acetonitrile to the working solutions ranging from 0.4 to 200 μg/L. The IS stock solution was prepared by dissolution in methanol. The IS working solution (40 μg/L) was prepared by diluting of the stock solution with acetonitrile. The ETV calibration standard was obtained by spiking blank plasma (50 μL), acetonitrile (50 μL) and deionized water (50 μL) with the appropriate working solutions (50 μL). Final concentrations of ETV in calibration samples were 0.5, 1, 2, 5, 10, 20 and 50 μg/L. Quality control (QC) samples at low (0.5 μg/L), medium (5 μg/L) and high (50 μg/L) concentrations were prepared separately in the same fashion. All the solutions were stored at 4 °C and brought to room temperature before use.

#### Sample preparation

Plasma samples (50 μL) were transferred to 1.5 mL centrifuge tubes and mixed with IS working solution (40 μg/L, 50 μL) before acetonitrile (100 μL) was added for protein precipitation. After vortexing for 1 min and centrifuging at 13,000 rpm for 10 min at 4 °C, the supernatant was transferred to labelled vials and a volume of 5 μL of this solution was immediately injected into UHPLC–MS/MS system for analysis.

#### Method validation

Selectivity was evaluated by comparing different batches (*n* = 5) of blank rat plasma with those plasma samples spiked with IS and plasma samples after oral administration of ETV to investigate potential interference at the LC peak region for ETV and IS. The linearity of the calibration curve containing seven non-zero concentrations was determined by plotting the peak area ratios of ETV to IS (analyte/IS, *y*) against the nominal concentrations of ETV using a least squares linear regression model with weighted (1/*x*^2^). The lower limit of quantification (LLOQ) was defined as the lowest possible drug concentration that was reproducibly detected. The signal-to-noise (S/N) ratio at this concentration was at least 10 times higher than that of blank plasma. Precision and accuracy were assessed by performing replicate analysis of QC samples (*n* = 5) at three concentrations of the calibration standards (0.5, 05 and 50 μg/L). This procedure was repeated using the plasma matrix on three different days to determine inter-day precision values. The accuracy of the assay was expressed as recovery of QC samples at the three concentrations following the addition of different amounts of ETV into blank plasma and extraction by protein precipitation. Apparent concentrations were calculated using the calibration curves. Extraction recovery was evaluated at three QC levels (*n* = 5) by comparing mean peak areas obtained from blank plasma samples spiked before extraction with those from blank plasma samples spiked after extraction. Matrix effects were measured via comparison of the peak responses of the ETV obtained from blank plasma samples spiked after extraction to those of pure standard solutions containing ETV at the same concentration. The stability of ETV in plasma was assessed by analyzing replicate samples (*n* = 5) spiked with standards at low, mid and high QC concentrations stored under four sets of conditions: room temperature for 4 h, in the autosampler at 10 °C for 24 h, three freeze–thaw cycles from –20 °C to 25 °C, and –20 °C for 2 weeks. ETV was considered stable if assay values were within the acceptable limits of ±15% relative standard deviation (RSD). The stability of the IS (10 μg/L) was evaluated using the same method.

#### Pharmacokinetic study

The validated method was applied to the pharmacokinetic study of ETV in normal and hepatic fibrotic rats after oral administration of ETV. Twenty-four normal rats were divided into four groups of six: ETV-N, EF-0, EF-1 and EF-2. The ETV-N group was administered ETV (0.9 mg/kg) by gastric perfusion; the EF-0 group was administered 0.9 mg/kg ETV and 0.55 g/kg FZHY simultaneously; the EF-1 group received 0.9 mg/kg ETV (p.o.) 1 h after the final FZHY (0.55 g/kg) administration; the EF-2 group received 0.9 mg/kg ETV (p.o.) 2 h after the final FZHY (0.55 g/kg) administration. Twelve model rats were divided into two groups of six: ETV-M and EF-2 (*n* = 6). The ETV-M group was administered ETV (0.9 mg/kg) by gastric perfusion; the EF-M-2 group received 0.9 mg/kg ETV (p.o.) 2 h after the final FZHY (0.55 g/kg) administration. All rats were housed individually under normal conditions. Animals were fasted overnight before the experiment and they had free access to water. Venous blood samples (approximately 0.2 mL) were collected from the periorbital sinus vein into heparinized tubes before administration (0 h) and at 0.033, 0.083, 0.25, 0.5, 0.75, 1, 2, 4, 6, 8, 12 and 24 h after dosing. The blood samples were centrifuged at 13,000 rpm for 10 min at 4 °C. The separated serum samples were frozen in polypropylene tubes at –20 °C until pharmacokinetic analysis of ETV.

#### Pharmacokinetic analysis

The main pharmacokinetic parameters (apparent volume of distribution (*V*_d_), clearance rate (CL), mean residence time (MRT), elimination rate (*K*_e_) and elimination half-life (*t*_1/2_)) were analyzed using the non-compartmental pharmacokinetics data analysis software of PK solutions 2™ (Summit Research Services, Montrose, CO). Peak plasma concentrations (*C*_max_) and the time to reach peak plasma concentration (*T*_max_) were obtained directly from the observed concentration versus time data. The areas under the plasma concentration versus time curves from zero to time *t* (AUC_0–_*_t_*) and from zero to infinity (AUC_0_*_–∞_*) were calculated using the trapezoidal rule and with extrapolation to infinity with *K*_e_.

### Statistical analysis

Data were presented as mean ± standard deviation (SD). Comparisons between two different groups at the same phase were conducted by the independent-samples *t*-test. All data analyses were performed with SPSS version 17.0 (SPSS, Chicago, IL). *p* Value of <0.05 was considered to indicate statistical significance.

## Results

### DMN-induced hepatic fibrosis

All rats in the model group were treated with DMN for 4 weeks. The liver sections of rats suffering from hepatic fibrosis (*n* = 6) were stained with H&E and Sirius Red to assess the degree of liver inflammation and fibrosis.

**Figure 2.**
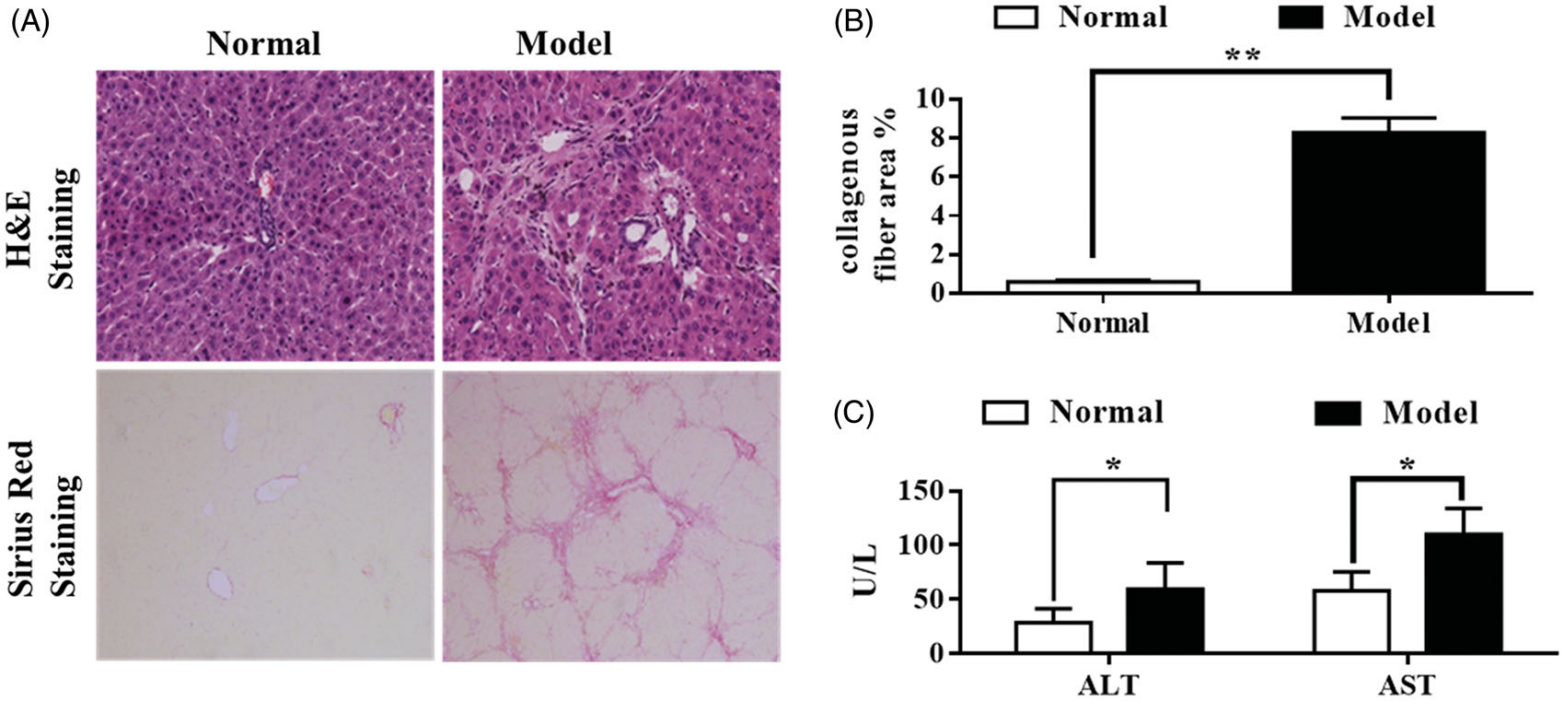
DMN-induced hepatic fibrosis in rats. Rats were injected with DMN (10 mg/kg body weight, i.p.) every other day for 4 weeks. (A) Staining with H&E (upper panels) or Sirius Red (lower panels) of liver samples from treated or not treated with DMN. (B) The collagenous fibres (A, lower panels) were quantified by imaging analysis. (C) Serum levels of ALT and AST of rats treated or not treated with DMN. *p < 0.05, **p < 0.01 compared with the normal group. Data are the mean ± SD (*n* = 6). Data were analyzed using Student’s *t*-test.

As expected, 4 weeks of DMN administration caused hepatic fibrosis in rats, as assessed by Sirius Red staining. DMN induced the synthesis of collagen, which was seen to stretch from portal to lobular areas. Complete septa and even regenerated nodules were also observed (Figure 2). The fibrosis was identified as stage 3 or 4. Therefore, the fibrosis model was shown to be suitable for use in pharmacokinetic studies.

### Method validation

**Figure 3.**
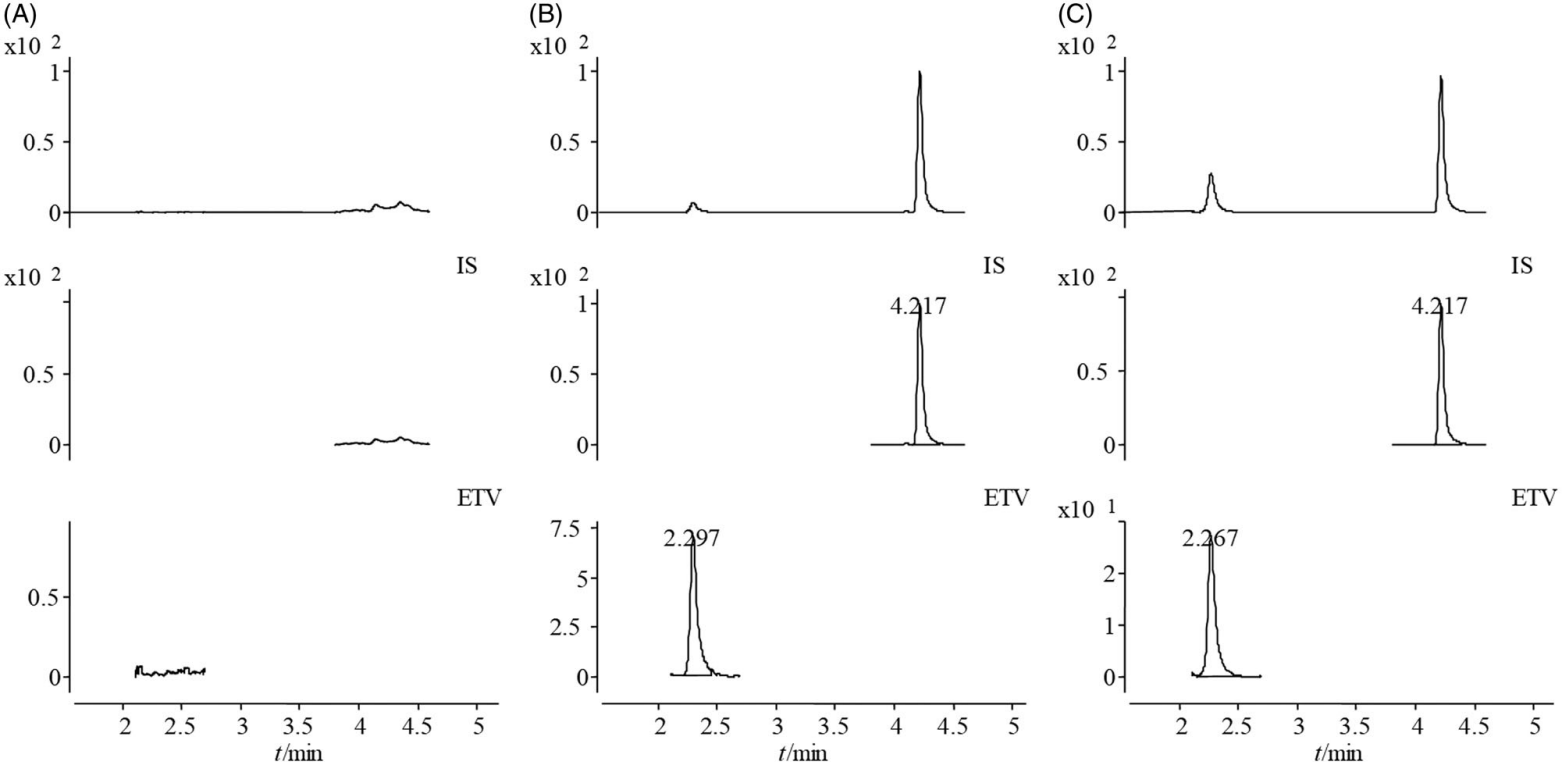
Typical multiple reaction monitoring (MRM) chromatograms of entecavir (*m/z* 277) and IS (*m/z* 229) separated on an ACQUITY UPLC HSS T3 column (2.1 mm × 100 mm, 1.8 μm) maintained at 40 °C and eluted with a gradient system composed of 0.1% formic acid in water and acetonitrile at a flow rate of 0.3 mL/min. A gradient programme was used as follows (time, min/acetonitrile %): 0/5, 2/15, 2.5/30, 3.2/90, 4.5/98, 4.6/5 and 6/5. (A) Blank serum sample; (B) blank plasma spiked with entecavir (500 μg/L); (C) rat plasma 30 min after oral administration of entecavir.

No interference from endogenous substances was observed in quantification transitions of ETV and IS. The representative mass chromatograms of blank plasma sample (A), plasma sample spiked with IS (10 μg/L) (B) and IS (10 μg/L) spiked plasma after oral administration of ETV (0.9 mg/kg) (C) are shown in Figure 3. The ETV calibration curve was linear over the concentration range 0.5–50 μg/L. The calibration curve and correlation coefficient (*r*) of ETV were *y* = 0.0265*x* – 0.0108 and *r* = 0.9996. The LLOQ of ETV was 0.5 μg/L (S/N> 10). The accuracy and precision data for the intra- and inter-day analysis are shown in Table 1. The RSD values of the precision of ETV detection (intra-day and inter-day) at three QC levels ranged between 5.02% and 9.91%. The mean accuracy of ETV detection was 104.18 ± 9.46%. These results demonstrated that both the accuracy and the precision of the present method were acceptable. The extraction recovery and matrix effect for ETV are shown in Table 1. The extraction recoveries of ETV at low, mid and high concentration levels (*n* = 5) were 99.48 ± 11.91%, 106.79 ± 3.35% and 120.53 ± 6.29%, respectively. These data indicated that the extraction recovery of ETV was efficient, consistent and reproducible. Matrix effects in the current method were also within acceptable limits (94.01–100.22%). Therefore, ion suppression and plasma enhancement effects were negligible for this method. The results of all stability tests are shown in Table 1. ETV was stable under the four sets of storage conditions (room temperature for 4 h, in the auto-sampler at 10 °C for 24 h, three freeze–thaw cycles from –20 °C to 25 °C, and –20 °C for 2 weeks). The SD about the mean of the test responses was within –3.5 to 0.63% in all the stability tests of QC samples. These stabilities met this need of a routine pharmacokinetic study.

**Table 1.** Summary of accuracy, precision, matrix effect, extraction recovery and stability of ETV determined using the UPLC–MS/MS method (data represent mean ± SD).

Spiked conc. (μg/L)	Measured conc. (μg/L)	Accuracy (%)	Mean accuracy (%)	Extraction recovery (%)	Matrix effect/%	Precision (RSD, %)		Stability (RSD %)			
						Inter-day	Intra-day	Room temperature (4 h)	Auto-sampler (10 °C, 24 h)	Freeze–thaw cycles	–20 °C (2 weeks)
0.5	0.56 ± 0.05	112.31 ± 10.75		99.48 ± 11.91	98.03 ± 12.57	5.02	7.83	3.22	7.34	8.13	3.14
10	5.01 ± 0.25	100.11 ± 5.08	104.18 ± 9.46	106.79 ± 3.35	94.01 ± 7.94	6.94	5.53	5.54	7.44	10.63	8.19
50	50.05 ± 6.27	100.10 ± 12.54		120.53 ± 6.29	100.22 ± 12.51	7.28	9.91	5.91	4.65	2.68	–3.56

### Pharmacokinetics of ETV in normal rats

**Figure 4.**
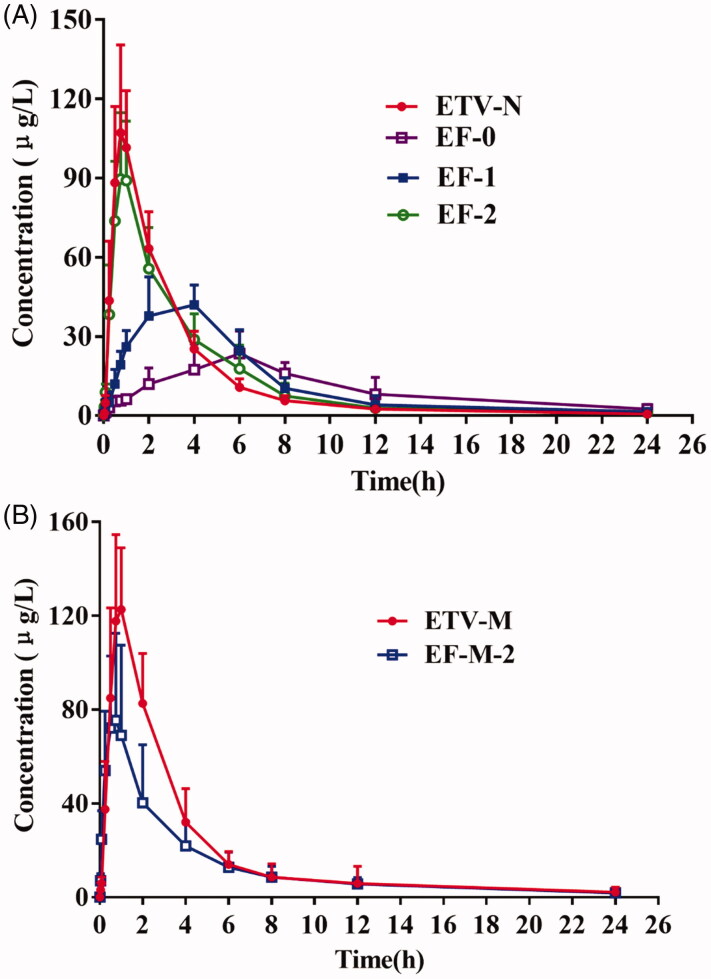
Plasma concentration–time curves of ETV (mean ± SD, *n* = 6) after oral administration of ETV alone (0.9 mg/kg) in (A) normal rats and (B) dimethylnitrosamine-induced hepatic fibrosis rats.

The ETV plasma concentrations profiles in normal rats after oral administration are shown in Figure 4. The estimated pharmacokinetic parameters of ETV are listed in Table 2, and the pharmacokinetic parameters of ETV in each rat are listed in Supplement Tables 1–6.

**Table 2.** The pharmacokinetic parameters of ETV after oral administration of ETV at a dose of 0.9 mg/kg alone in different groups (data represent mean ± SD).

Parameters	Normal rats (*n* = 6)				Hepatic fibrosis rats	
	ETV-N	EF-0	EF-1	EF-2	ETV-M (*n* = 6)	EF-M-2 (*n* = 5)
*C*_max_ (μg/L)	110.85 ± 24.25	27.38 ± 7.52**	42.43 ± 3.15**	101.37 ± 20.54	127.72 ± 29.48	76.54 ± 36.16^#^
*T*_max_ (h)	0.75 ± 0.16	6.00 ± 1.26**	3.67 ± 0.82**	0.87 ± 0.10	0.88 ± 0.14	0.68 ± 0.16^#^
*K*_e_ (1/h)	0.14 ± 0.03	0.17 ± 0.06	0.10 ± 0.02**	0.13 ± 0.05	0.09 ± 0.02**	0.09 ± 0.02
*t*_1/2e_ (h)	4.96 ± 0.90	4.50 ± 1.46	7.41 ± 1.22**	6.04 ± 1.91	8.01 ± 1.30**	7.91 ± 1.89
AUC_0–_*_t_* (μg·h/L)	323.84 ± 44.63	236.67 ± 48.91*	281.67 ± 57.38	356.85 ± 83.49	437.61 ± 130.14	306.12 ± 145.93
AUC_0–∞_ (μg·h/L)	328.54 ± 44.96	244.41 ± 51.25	301.18 ± 73.00	365.22 ± 79.43	464.65 ± 155.28	327.06 ± 146.28
MRT (h)	3.77 ± 0.37	8.42 ± 1.38**	7.31 ± 2.23**	4.52 ± 1.08	5.66 ± 2.37	7.40 ± 1.51
*V*_d_/*F* (L/kg)	19.83 ± 4.30	25.18 ± 10.73*	3.27.±5.51**	2.31 ± 10.47	24.38 ± 9.10*	38.09 ± 21.85
CL/*F* (L/h/kg)	2.78 ± 0.33	3.84 ± 0.91*	3.12±.067	2.55 ± 0.48	2.101 ± 0.64	3.14 ± 1.14

* *p* < 0.05, **p < 0.01 compared with ETV group. #*p* < 0.05 compared with ETV-M group.

The mean (SD) plasma concentration time profiles of ETV after intragastric administration of ETV in normal rats are shown in Figure 4(A). A graph using a log–linear scale is shown in Supplementary Figure. ETV was absorbed rapidly following intragastric administration of a dose of 0.9 mg/kg, with *C*_max_ occurring, in general, at 0.75 h. Drug deposition appeared to be rapid, with mean plasma concentrations falling to ≤10% of the mean *C*_max_ 24 h after dosing. Comparatively speaking, the ETV plasma concentration–time curve in the EF-2 group (Figure 4(A)) was similar to that in the ETV-N group, with no statistically significant differences in the pharmacokinetic parameters between the two groups. In contrast, the ETV plasma concentration–time curve in the EF-0 group increased slowly to a maximum value at 6 h, and then declined slowly to 1440 min (Figure 4(A)). There were no significant differences in the *K*_e_ and *t*_1/2e_ values for ETV between the ETV-N and EF-0 groups. However, the ETV *C*_max_ value was only 27.38 ± 7.52 μg/L in the EF-0 group, while it was 110.85 ± 24.25 μg/L in the ETV group, and the AUC_0–_*_t_* value (323.84 ± 44.63 μg·h/L) in the EF-0 group was approximately 0.73-fold less than that in the ETV-N group (236.67 ± 48.91 ng/h/mL). In addition, the *T*_max_ and MRT values were increased to 6.00 ± 1.26 h and 8.42 ± 1.38 h, respectively. The *V*_d_ and CL values were significantly increased to 25.18 ± 10.73 L/kg (*p* < 0.05) and 3.84 ± 0.91 L/h/kg (*p* < 0.05). In summary, the pharmacokinetics of ETV were altered significantly by co-administration with FZHY, as shown by a decrease in *C*_max_ of 75.30%, a decrease in AUC_0–_*_t_* of 26.92% and a delay in *T*_max_ of 5.25 h. The pharmacokinetics profiles of ETV in the EF-1 group are shown in Figure 4(A). The ETV plasma concentration–time curve produced a peak at 4 h and then declined. Compared to the ETV-N group, the *C*_max_ value in the EF-1 group was significantly lower at 42.43 ± 3.15 μg/L (*p* < 0.01), while the *V*_d_ value increased to 32.71 ± 5.51 mL/kg (*p* < 0.01). In addition, the *T*_max_ (3.67 ± 0.82 h), *t*_1/2e_ (7.41 ± 1.22 h) and MRT (7.31 ± 2.23 h) values of ETV were significantly increased compared with the values in the ETV-N group.

### Pharmacokinetics of ETV in dimethylnitrosamine-induced hepatic fibrosis rats

The ETV plasma concentration profiles in DMN-induced hepatic fibrosis rats after oral administration of ETV are shown in Figure 4(B). The estimated pharmacokinetic parameters of ETV are listed in Table 2.

There was no significant difference in the plasma concentration–time curve following intragastric administration of ETV to normal and DMN-induced hepatic fibrosis rats (Figure 4(B)). Furthermore, there were no significant differences in the pharmacokinetic parameters of ETV (*C*_max_, *T*_max_, AUC_0–_*_t_*, AUC_0–∞_, MRT and CL) measured under the normal physiological and pathological conditions (Table 2). However, in the ETV-M groups, the *t*_1/2e_ and *V*_d_/*F* were increased significantly to 8.01 ± 1.30 h (*p* < 0.01) and 24.38 ± 9.10 L/kg (*p* < 0.05) compared with that of the ETV-N group, respectively. Otherwise, in the EF-M-2 group, the *t*_1/2e_ (7.91 ± 1.89 h) and MRT (7.40 ± 1.51 h) values were increased compared with that of the ETV-N group. The ETV plasma concentration–time curve in the EF-M-2 group rats was similar to that in the ETV-M group. Although the *C*_max_ of ETV was significantly lower (76.54 ± 36.16 μg/L) in model rats compared with that in normal rats (127.72 ± 29.48 μg/L, *p* < 0.05*)* and its *T*_max_ was more rapid (*p* < 0.05*)*, there were no significant differences in other parameters.

## Discussion

In this study, a fast and sensitive UHPLC–MS/MS technique was successfully applied to the determination of ETV in the plasma of rats following administration alone or in combination with FZHY. Compared with the solid-phase extraction (SPE) pre-treatment method used by Zhao et al. (2012), we established a simple, sensitive, rapid and economical method based on protein-precipitation pre-treatment. This method could be used to remove most endogenous substances and obtain a high recovery rate. The matrix effect met the requirement of biological analyses.

FZHY could significantly reduce the *C*_max_ and AUC_0–_*_t_*, and significantly increase the *T*_max_, MRT and *V*_d_ of ETV, if ETV was administered simultaneously with FZHY. The effect of FZHY on the pharmacokinetics of ETV was manifested mainly in absorption. However, the influence of FZHY on ETV pharmacokinetics was gradually reduced with the prolongation of intervals between them. The pharmacokinetics of ETV were virtually unaffected if the interval of administration was 2 h. Conversely, rats who had suffered DMN-induced hepatic fibrosis could prolong the *t*_1/2_ of ETV, suggesting that the elimination was slowed down. Although the FZHY interval was 2 h, the *t*_1/2_ of ETV was prolonged, and the *C*_max_ and *T*_max_ of ETV decreased significantly.

Our data could have been the result of three main effects. First, the drug–time curve suggested that the effect of FZHY on the pharmacokinetics of ETV was like that seen for food (Matthews 2006). Under this circumstance, FZHY could inhibit ETV absorption like that seen for food. Second, the potential drug–drug interactions of ETV are mediated by transporters. ETV is the substrate of OAT1, OAT3 and OCTs (Mandíková et al. 2016). The active compounds of FZHY are transported through the small intestine after mediation by OATs such as schisandrin A, angeloylgomisin M1, schisandrol B, tigloylgomisin H and schisantherin A (Pan et al. 2006; Xu et al. 2013). If FZHY and ETV are administered simultaneously, FZHY could occupy absorption sites, leading to inhibition of ETV absorption. Two-hour intervals avoided competitive absorption between the two drugs, but the mechanism of action merits further study. Finally, the effects of DMN on ETV pharmacokinetics are not known. Therefore, more factors may need be considered to explain the effects of FZHY on ETV pharmacokinetics in the DMN-induced model group.

Our study had two main limitations. First, we studied only the effects of FZHY on ETV pharmacokinetics, and did not clarify its mechanism of action. Second, the animal model is quite different from the fibrosis caused by CHB infection, which may not reflect exactly the clinical situation. Therefore, more research must be carried out to clarify the interaction between ETV and FZHY.

## Conclusions

The effects of FZHY on ETV pharmacokinetics were diminished with an increase in interval time. ETV pharmacokinetics changed slightly in rats with DMN-induced hepatic fibrosis. We suggest that ETV should be taken in the absence of FZHY for optimal bioavailability.

## Supplementary Material

05-supplementry_table_and_figure.docx
