# Supplementary material for: Effects of Fuzheng Huayu recipe on entecavir pharmacokinetics in normal and dimethylnitrosamine-induced hepatic fibrosis rats
Source: Pharm Biol. 2019 Dec 18;58(1):1–7. doi: 10.1080/13880209.2019.1687527 (PMC6968529; doi:10.1080/13880209.2019.1687527)
Supplement: 05-supplementry_table_and_figure.docx [file IPHB_A_1687527_SM0454.docx]

**Supplement table and figure**

Table 1 The pharmacokinetic parameters of ETV for each rat after oral administration ETV at dose of 0.9 mg/kg in ETV-N groups.

| Parameters | **1** | **2** | **3** | **4** | **5** | **6** | mean | SD |
| --- | --- | --- | --- | --- | --- | --- | --- | --- |
| *K*_e_(1/h) | 0.13 | 0.18 | 0.19 | 0.12 | 0.12 | 0.13 | 0.14 | 0.03 |
| *t*_1/2e_(h) | 5.43 | 3.89 | 3.72 | 5.59 | 5.58 | 5.53 | 4.96 | 0.90 |
| *C*_max_(μg/L) | 91.72 | 84.47 | 111.75 | 154.17 | 111.59 | 111.36 | 110.85 | 24.25 |
| *T*_max_(h) | 0.50 | 1.00 | 0.75 | 0.75 | 0.75 | 0.75 | 0.75 | 0.16 |
| *AUC*_(0-t)_(μg·h/L) | 275.18 | 316.63 | 324.86 | 408.08 | 306.99 | 311.27 | 323.84 | 44.63 |
| *AUC*_(0-∞)_(μg·h/L) | 282.64 | 318.67 | 326.05 | 415.07 | 312.49 | 316.30 | 328.54 | 44.96 |
| *MRT*(h) | 4.37 | 3.92 | 3.30 | 3.83 | 3.55 | 3.65 | 3.77 | 0.37 |
| *V*_d_/F(L/kg) | 24.96 | 15.84 | 14.81 | 17.48 | 23.19. | 22.69. | 19.83.4 | 4.30 |
| *CL*/F (L/h/kg) | 3.18. | 2.8.2 | 2.76 | 2.17 | 2.88. | 2.85 | 2.78 | 0.33 |

Table 2 The pharmacokinetic parameters of ETV for each rat after oral administration ETV at dose of 0.9 mg/kg in EF-0 groups.

| Parameters | **1** | **2** | **3** | **4** | **5** | **6** | mean | SD |
| --- | --- | --- | --- | --- | --- | --- | --- | --- |
| *K*_e_(1/h) | 17.84 | 24.12 | 30.97 | 36.45 | 34.02 | 20.90 | 27.38 | 7.52 |
| *t*_1/2e_(h) | 6.00 | 6.00 | 4.00 | 6.00 | 6.00 | 8.00 | 6.00 | 1.26 |
| *C*_max_(μg/L) | 0.13 | 0.13 | 0.11 | 0.17 | 0.23 | 0.24 | 0.17 | 0.06 |
| *T*_max_(h) | 5.20 | 5.50 | 6.44 | 4.05 | 2.95 | 2.83 | 4.50 | 1.46 |
| *AUC*_(0-t)_(μg·h/L) | 157.57 | 286.78 | 233.86 | 217.04 | 235.19 | 289.57 | 236.67 | 48.91 |
| *AUC*_(0-∞)_(μg·h/L) | 164.86 | 305.53 | 245.03 | 220.97 | 236.14 | 293.93 | 244.41 | 51.25 |
| *MRT*(h) | 8.86 | 9.67 | 7.64 | 7.97 | 6.34 | 10.02 | 8.42 | 1.38 |
| *V*_d_/F(L/kg) | 4.10. | 23.39 | 34.14 | 23.82 | 16.24 | 12.52 | 25.182 | 10.732 |
| *CL*/F (L/h/kg) | 5.46 | 2.95 | 3.67 | 4.07 | 3.81 | 3.06 | 3.83 | 0.91 |

Table 3 The pharmacokinetic parameters of ETV for each rat after oral administration ETV at dose of 0.9 mg/kg in EF-1 groups.

| Parameters | **1** | **2** | **3** | **4** | **5** | **6** | mean | SD |
| --- | --- | --- | --- | --- | --- | --- | --- | --- |
| *K*_e_(1/h) | 0.10 | 0.12 | 0.08 | 0.10 | 0.08 | 0.08 | 0.10 | 0.02 |
| *t*_1/2e_(h) | 6.67 | 5.68 | 8.45 | 6.73 | 8.61 | 8.36 | 7.41 | 1.22 |
| *C*_max_(μg/L) | 37.62 | 39.30 | 44.59 | 43.79 | 44.99 | 44.29 | 42.43 | 3.15 |
| *T*_max_(h) | 4.00 | 4.00 | 4.00 | 2.00 | 4.00 | 4.00 | 3.67 | 0.82 |
| *AUC*_(0-t)_(μg·h/L) | 223.96 | 253.25 | 352.55 | 250.48 | 356.10 | 253.66 | 281.67 | 57.38 |
| *AUC*_(0-∞)_(μg·h/L) | 229.68 | 264.63 | 373.61 | 259.84 | 411.34 | 267.96 | 301.18 | 73.00 |
| *MRT*(h) | 5.63 | 7.18 | 7.65 | 5.65 | 11.53 | 6.20 | 7.31 | 2.23 |
| *V*_d_/F(L/kg) | 37.69 | 27.85 | 29.37 | 33.63 | 27.19 | 40.51 | 32.71 | 5.50 |
| *CL*/F (L/h/kg) | 3.92 | 3.40 | 2.41 | 3.46 | 2.19 | 3.36 | 3.12 | 0.67 |

Table 4 The pharmacokinetic parameters of ETV for each rat after oral administration ETV at dose of 0.9 mg/kg in EF-2 groups.

| Parameters | **1** | **2** | **3** | **4** | **5** | **6** | mean | SD |
| --- | --- | --- | --- | --- | --- | --- | --- | --- |
| *K*_e_(1/h) | 0.17 | 0.06 | 0.10 | 0.11 | 0.10 | 0.08 | 0.10 | 0.04 |
| *t*_1/2e_(h) | 3.97 | 11.59 | 6.88 | 6.44 | 6.78 | 8.58 | 7.37 | 2.54 |
| *C*_max_(μg/L) | 81.31 | 79.90 | 101.04 | 95.93 | 131.94 | 79.90 | 95.00 | 20.23 |
| *T*_max_(h) | 1.00 | 0.75 | 1.00 | 0.75 | 0.75 | 0.75 | 0.83 | 0.13 |
| *AUC*_(0-t)_(μg·h/L) | 372.04 | 283.78 | 284.85 | 343.47 | 354.48 | 277.78 | 319.40 | 41.89 |
| *AUC*_(0-∞)_(μg·h/L) | 375.18 | 327.29 | 292.64 | 352.64 | 361.67 | 297.62 | 334.51 | 34.31 |
| *MRT*(h) | 4.00 | 9.68 | 4.11 | 4.71 | 3.56 | 6.63 | 5.45 | 2.33 |
| *V*_d_/F(L/kg) | 13.73 | 45.98 | 30.51 | 23.71 | 24.36 | 37.45 | 29.29 | 11.35 |
| *CL*/F (L/h/kg) | 2.40 | 2.75 | 3.08 | 2.55 | 2.49 | 3.02 | 2.71 | 0.28 |

Table 5 The pharmacokinetic parameters of ETV for each rat after oral administration ETV at dose of 0.9 mg/kg in ETV-M groups.

| Parameters | **1** | **2** | **3** | **4** | **5** | **6** | mean | SD |
| --- | --- | --- | --- | --- | --- | --- | --- | --- |
| *K*_e_(1/h) | 0.10 | 0.08 | 0.08 | 0.11 | 0.09 | 0.07 | 0.09 | 0.02 |
| *t*_1/2e_(h) | 6.78 | 8.92 | 8.93 | 6.22 | 7.79 | 9.44 | 8.01 | 1.30 |
| *C*_max_(μg/L) | 149.20 | 87.54 | 147.20 | 93.79 | 134.54 | 154.05 | 127.72 | 29.48 |
| *T*_max_(h) | 0.75 | 1.00 | 1.00 | 1.00 | 0.75 | 0.75 | 0.88 | 0.14 |
| *AUC*_(0-t)_(μg·h/L) | 525.20 | 276.01 | 401.47 | 415.97 | 361.30 | 645.69 | 437.61 | 130.14 |
| *AUC*_(0-∞)_(μg·h/L) | 536.51 | 289.50 | 417.80 | 427.49 | 379.96 | 736.63 | 464.65 | 155.28 |
| *MRT*(h) | 3.88 | 5.20 | 4.54 | 4.74 | 5.20 | 10.40 | 5.66 | 2.37 |
| *V*_d_/F(L/kg) | 16.42 | 40.00 | 27.75 | 18.89 | 26.61 | 16.64 | 24.38 | 9.10 |
| *CL*/F (L/h/kg) | 1.68 | 3.11 | 2.15 | 2.11 | 2.37 | 1.22 | 2.11 | 0.64 |

Table 6 The pharmacokinetic parameters of ETV for each rat after oral administration ETV at dose of 0.9 mg/kg in EF-M-2 groups.

| Parameters | **1** | **2** | **3** | **4** | **5** | mean | SD |
| --- | --- | --- | --- | --- | --- | --- | --- |
| *K*_e_(1/h) | 0.11 | 0.07 | 0.11 | 0.07 | 0.10 | 0.09 | 0.02 |
| *t*_1/2e_(h) | 6.35 | 9.74 | 6.33 | 10.17 | 6.94 | 7.91 | 1.89 |
| *C*_max_(μg/L) | 72.71 | 74.46 | 137.26 | 46.51 | 51.74 | 76.53 | 36.13 |
| *T*_max_(h) | 0.50 | 0.75 | 0.75 | 0.50 | 0.75 | 0.65 | 0.14 |
| *AUC*_(0-t)_(μg·h/L) | 311.41 | 208.58 | 546.11 | 172.46 | 292.00 | 306.11 | 145.95 |
| *AUC*_(0-∞)_(μg·h/L) | 326.79 | 229.44 | 570.36 | 198.41 | 310.28 | 327.06 | 146.25 |
| *MRT*(h) | 6.55 | 7.28 | 5.90 | 9.93 | 7.34 | 7.40 | 1.53 |
| *V*_d_/F(L/kg) | 25.25 | 55.15 | 14.42 | 66.58 | 29.04 | 38.09 | 21.85 |
| *CL*/F (L/h/kg) | 2.75 | 3.92 | 1.58 | 4.54 | 2.90 | 3.14 | 1.14 |

**Supplement figure**


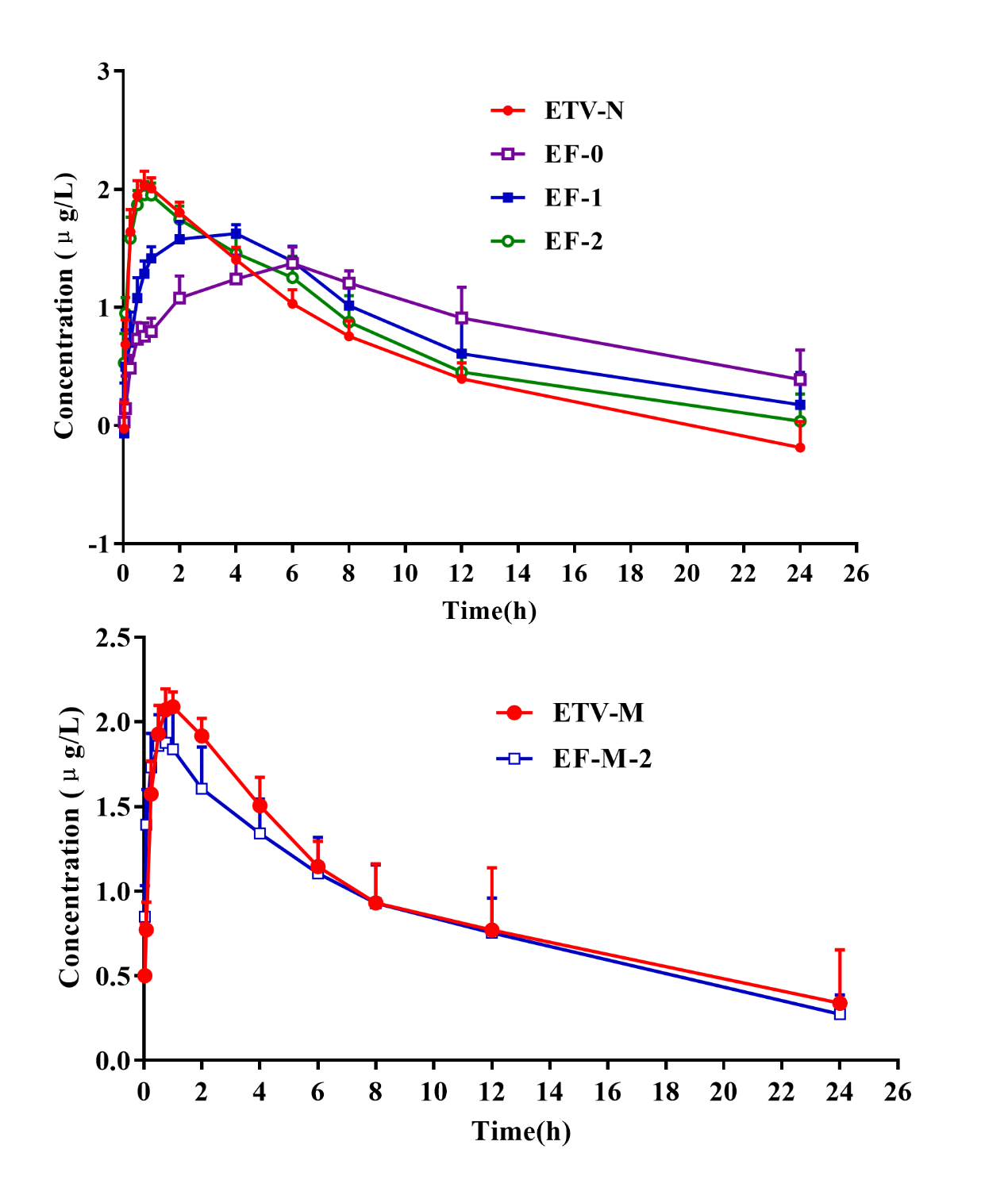


**Supplement figure 1** Plasma concentration-time curves of ETV (mean ± SD, n = 6) after oral administration of ETV alone (0.9 mg/kg) in (A) normal rats and (B) dimethylnitrosamine-induced hepatic fibrosis rats. The scale of concentration have been log-linear scale(lgCi).
